# The hope for targeting fatty acid binding proteins in multiple myeloma

**DOI:** 10.18632/oncotarget.28437

**Published:** 2023-06-19

**Authors:** Heather Fairfield, Michaela R. Reagan

**Keywords:** multiple myeloma, FABPs, fatty acid binding proteins, immunoncology, pre-clinical discovery

The proteins that comprise the fatty acid binding protein family, known as FABPs, have recently been implicated as prognostic markers identifying more aggressive myeloma cells, as we reported earlier this year in *eLife* [1]. The FABPs hold promise as new therapeutic targets in multiple myeloma (MM), as described by our laboratory, and supported by *in silico* analyses [2] and other data [3, 4]. In multiple datasets, (including GSE4452, GSE4204, GSE132604, GSE4452, GSE9782, and the MMRF’s CoMMpass dataset) progression free survival and overall survival were worse for patients with high tumor cell expression of *FABP5*. Similarly *in vitro*, we found that myeloma cell number was dramatically decreased by FABP inhibitors, which increased tumor cell apoptosis and decreased tumor cell proliferation [1]. The mechanisms of action of FABP-driven support of myeloma appear to be plentiful. Using proteomics and RNA-seq analysis, we found that inhibition of the FABP family in myeloma cells led to changes such as a decrease in Ki-67 expression, downregulation of pathways related to the unfolded protein response and ER stress responses, as well as an increase in reactive oxygen species. FABP inhibition also negatively impacted expression of *MYC* and the Myc pathway, which is key for myeloma cell survival and proliferation. In addition, mitochondrial membrane potential, oxygen consumption rate, fatty acid oxidation, and DNA methylation properties were also altered by FABP inhibition [1].

Jia et al. also found differences in immune-related mRNA expression between MM patient bone marrow (BM) and normal BM, and observed that *FABP5* expression in MM cells correlates with immune cell changes in the tumor microenvironment [2]. These data suggest that *FABP5* expression in MM cells may also influence tumor progression by affecting the infiltrating BM immune cells. Data from Dr. Liang’s laboratory presented at two American Society of Hematology conferences, also suggest that FABPs are important in MM [3, 4]. They reported increased FABP4 expression in MM patients versus healthy controls and a correlation between serum FABP4 levels and M protein levels in MM human patients. In addition, when U266 human myeloma cells were injected into mice with global knockout of *FABP4,* KO mice exhibited improved survival, less tumor burden, and fewer MM-related osteolytic lesions compared to wild type controls, when both groups were on a high fat diet. FABP4-KO mice also had differences in immune cells and bone marrow cytokine levels. Lastly, they found that treatment with a FABP inhibitor decreased tumor burden in obese mice, compared to vehicle. Their exciting findings further support the potential of FABP inhibition in MM, and show that FABPs from the microenvironment, not only those expressed by MM cells, should be targeted. Hopefully the full details of this work, including details of the patient samples used, mice (sex, strain, age, weight gain) and tumor models will be available soon. It is possible that increased *CCL3*, which attracts macrophages, monocytes and neutrophils, and which we observed in MM cells treated with FABP inhibitors, could induce these changes in immune cell infiltration. We reported studies showing either decreased tumor burden or no effect of FABP inhibition *in vivo*, and thus further optimization of *in vivo* targeting of FABPs, FABP inhibitor design, or overcoming FABP inhibitor resistance in the bone marrow is still required before translation to the clinic can materialize [1]. Still, we are hopeful that by targeting FABPs, or following the science to other related pathways, it will be possible to revolutionize the therapy regimes currently used for MM patients.
